# Trustful relationships between healthcare professionals and children: a concept analysis using Rodgers’ evolutionary approach

**DOI:** 10.1007/s00431-025-06297-0

**Published:** 2025-07-03

**Authors:** Rhymme Dickens, Piet Leroy, Walter Eppich, Maria Brenner

**Affiliations:** 1 School of Health Professions Education, Faculty of Health, Medicine and Life Sciences, Maastricht University, Maastricht, The Netherlands; 2 Faculty of Medicine, Dentistry and Health Sciences, University of Melbourne, Victoria, 3010 Australia; 3 School of Nursing, Midwifery and Health Systems, Health Sciences Building, University College Dublin, Belfield, Dublin 4, Ireland

**Keywords:** Trustful relationships, Trusting relationships, Children, Healthcare professional, Rapport building, Concept analysis

## Abstract

Despite the recognized importance of trust in pediatric healthcare, the concept of “trustful relationships” between children and healthcare professionals (HCPs) remains poorly defined, leading to inconsistencies in clinical practice and education. This study aims to define and clarify the concept of “trustful relationships” between pediatric patients aged 2 to 12 years and HCPs. It seeks to identify key attributes, antecedents, and consequences of these relationships and to develop a conceptual framework for future research and practice. An evolutionary concept analysis (ECA) following Rodgers’ (2000) framework was conducted to explore the dynamic nature of trust in pediatric care. A comprehensive literature search was performed using PubMed, Embase, CINAHL, and PsychINFO databases, covering publications from 1936 until 2024. Forty articles meeting inclusion criteria were analyzed. The analysis identified surrogate terms such as “trusting relationship” and “established rapport.” Fifty-six attributes of trustful relationships emerged, including HCP characteristics, time, language, and non-verbal communication. Twenty-three antecedents were identified, such as preparation, parental presence, and environmental factors. Additionally, 22 consequences were recognized, encompassing both emotional outcomes (e.g., reduced anxiety, increased comfort) and medical outcomes (e.g., improved adherence to treatment plans).

*Conclusion:* This study highlights the complexity of trustful relationships between children and HCPs, emphasizing the importance of empathy, effective communication, and a child-centered environment. Integrating these findings into healthcare education and clinical practice can improve pediatric care by fostering stronger provider-patient relationships, reducing patient anxiety, and enhancing health outcomes.

**Table:** 

**What is known:** • *Trust between children and HCPs in pediatric medicine has come to play a much larger role in recent years. However, the concept of “trustful relationships” between children and HCPs has not been systematically and empirically analyzed before, leaving it ill-defined and confusing.*
**What is new:** • *This is, to our knowledge, the first time this concept has been analyzed in a systematic and empirical way, and it provides a preliminary theoretical framework for better understanding the character of trustful relationships between children and HCPs.* • *Based on our findings, we propose a definition of trustful relationships between children and HCPs as “dynamic, reciprocal connections characterized by mutual curiosity, respect, and confidence. These relationships form the foundation of effective clinical care and develop through the interplay of contextual factors and professional behaviors intended to establish trust.”*

**Supplementary Information:**

The online version contains supplementary material available at 10.1007/s00431-025-06297-0.

## Introduction

Trust represents a fundamental cornerstone of healthcare delivery, forming the foundation upon which effective therapeutic relationships are built. In adult medicine, several efforts have been made to systematically and empirically synthesize the literature on trust, leading to greater conceptual clarity and a better understanding of its role in patient-provider interactions [1–3]. These reviews highlight that multiple definitions of trust exist, reflecting various theoretical perspectives and contexts. However, at its core, trust is commonly understood as “an individual’s belief that others (i.e. individuals as well as institutions) will act appropriately and perform competently, responsibly, and consider their personal interests” [4].

In contrast, pediatric healthcare has not yet benefited from a comparable synthesis of the literature on trust. This is a significant gap, given that the development and maintenance of trustful relationships between children and healthcare professionals (HCPs) are important. Trust supports effective communication, cooperation, and shared decision-making [5, 6]. When children trust their HCPs, they are more likely to engage meaningfully in their care, resulting in better health outcomes and higher levels of satisfaction [7–9]. Trust also helps to reduce fear and anxiety during medical encounters, which can facilitate physical examinations and procedures [6, 10–12].

Despite the widely acknowledged importance of establishing trustful relationships between children and HCPs, the concept itself is poorly defined, and we currently lack a comprehensive definition. The absence of a clear and broadly accepted definition may lead to confusion about the topic’s exact meaning, its correct application in clinical practice, and its integration into health professional education and clinical research. A well-defined understanding of the concept would assist practitioners, educators, and researchers in developing consistent strategies to establish trust in clinical practice, designing effective education, and fostering a high-quality research agenda.

This study aims to define and clarify the concept of “trustful relationships” between pediatric patients aged 2 to 12 years and HCPs. It seeks to identify key attributes, antecedents, and consequences of these relationships and to develop a conceptual framework for future research and practice. We conducted an evolutionary concept analysis (ECA) following Rodgers’ (2000) framework to explore the dynamic nature of trust in pediatric care. The resulting framework may serve as a foundation for future research and the development of a more comprehensive explanatory model for trustful pediatric patient-provider relationships [13].

## Methods

We chose ECA as the most appropriate methodology as it allows us to examine trust as a dynamic and context-dependent concept that evolves over time. This approach is well-suited for capturing the complexity and changing nature of trust in pediatric healthcare, providing insights into its attributes, antecedents, and consequences in varying contexts. A concept analysis (CA) aims to establish a comprehensive definition and enhance understanding of the concept being studied [13]. According to Rodgers, a clearly defined concept can be used more effectively and evaluated in terms of its strengths and weaknesses. Variations can be introduced and tested to refine the concept and make it more relevant to its contemporary context. Considering the dynamic nature of trustful relationships and children’s healthcare on a global scale, Rodgers’ ECA framework is particularly appropriate for exploring trustful relationships between healthcare professionals and children. Therefore, for this CA, we adopt Rodgers’ framework as the guiding methodology to clarify and analyze the concept of “trustful relationships” between pediatric patients and HCPs. Using this approach, we reviewed the scientific literature following these essential steps: (1) identifying and naming the concept of interest; (2) identifying its surrogate terms; (3) recognizing and selecting the appropriate realm for data collection; (4) collecting the relevant data to identify the attributes of the concept; (5) analyzing the data to find references, antecedents, and consequences of the concept; (6) identifying concepts related to the concept of interest; and (7) finding a model case of the concept of trustful relationships in the pediatric healthcare setting [13].

## Initial surrogate terms, data sources, and search strategy

Rodgers argues that a strong search strategy ensures a meticulously selected sample of the literature, represents the literature fairly, and ultimately reduces researcher bias [13]. A search strategy was developed in close cooperation with a university librarian to ensure its quality and completeness. Initial surrogate terms were identified through our reading of the background literature and were discussed within the research team. Mindful of our view that the area of exploration was trustful relationships leading to the agreement of the key search words for the concept of trustful relationships in pediatric healthcare, like “trust,” “health personnel,” and “children.” Exclusion criteria were age under 2 or above 12, articles focused on palliative care, pathophysiology, or child abuse. Articles about children with intellectual disabilities were not included, as well as children with syndromic or psychiatric disorders.

The term “trusting relationship” has been previously established in the literature [10, 14–19]. However, in this study, we deliberately use the term “trustful relationships” to emphasize a more profound and holistic interpretation of trust in the context of healthcare interactions. Although the Cambridge Dictionary does not differentiate between “trusting” and “trustful,” we argue that “trustful” more accurately conveys a relationship that is full of trust, not merely one in which individuals choose to trust, but one in which trust is embedded in the very fabric of the relationship [20]. A trustful relationship, as we define it, represents an all-encompassing philosophy of care. It begins at the very first encounter between a child, their family, and the healthcare system, and it shapes every subsequent interaction. This concept goes beyond mutual trust; it reflects the whole healthcare environment.

## Data extraction and analysis

**Fig. 1:**
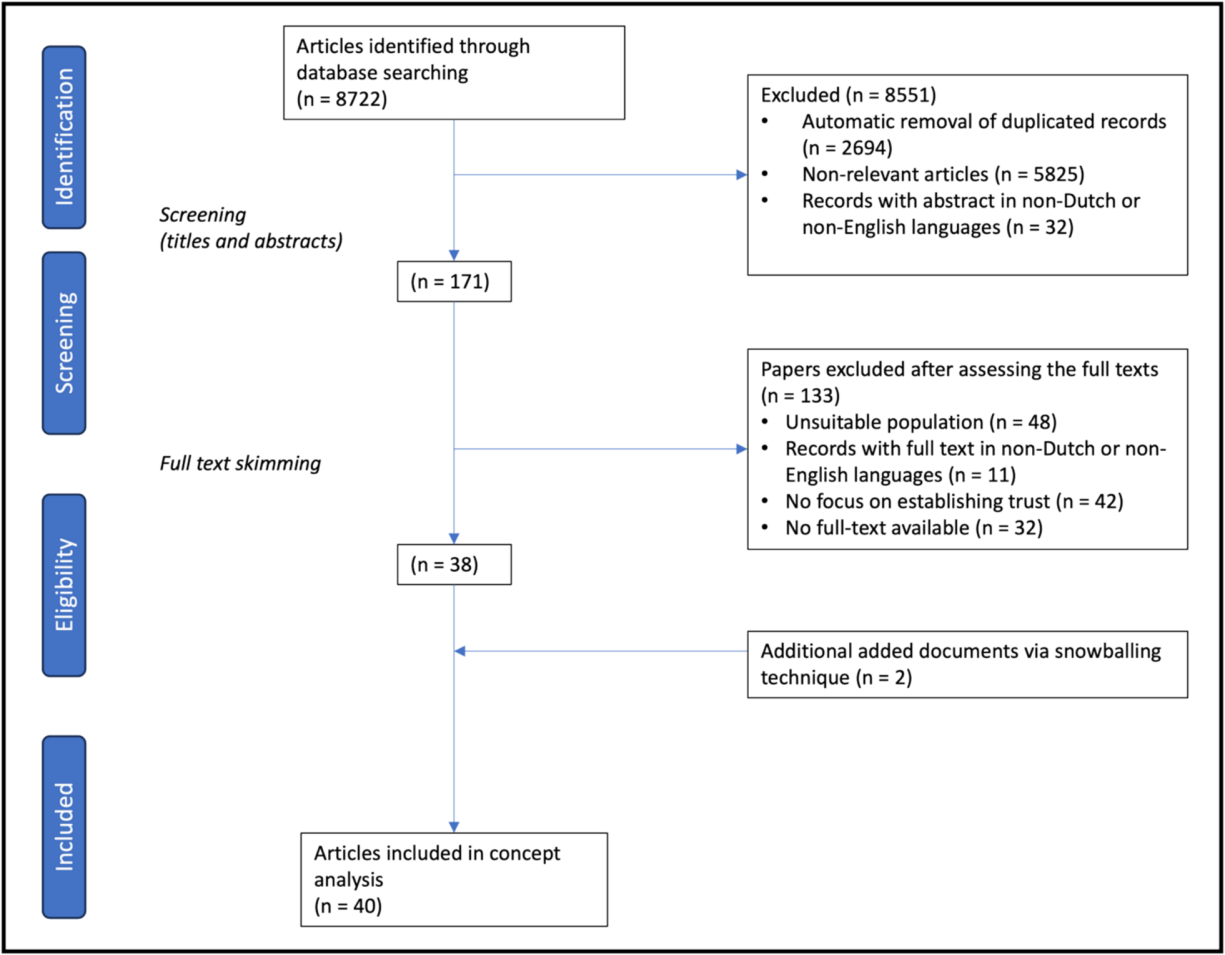
Summary of the search

We searched the literature using several databases (PubMed, Embase, PsycINFO, and CINAHL), yielding 8722 articles, of which 2694 duplicates were removed by Endnote Software. The titles and abstracts of the remaining 6028 articles were screened, after which another 5857 articles were excluded for lack of relevance or foreign language. The full text of the remaining 171 articles was reviewed by the lead researcher (RD) and second author (MB). The review of these residuary articles resulted in the exclusion of 133 articles for the following reasons: abstracts only, different research populations, or not focused on establishing trust. Two further articles were identified in the process of this full-text review, after snowballing technique. This led us to the 40 articles included in this CA (Fig. 1). The search included literature published up to 2024, and the final selection of articles spanned from 1936 to 2024.

In a CA, data analysis aims to identify a consensus in the existing literature [13]. The data was carefully examined for areas of agreement and disagreement, changes over time, and emerging trends that indicate needs for further research. Once all relevant data was identified, each item was read repeatedly to ensure all relevant data was extracted and a general tone was obtained. Data extraction records were maintained for each article, and information relevant to each of the major categories was recorded separately, including surrogate terms, related concepts, attributes, antecedents, and consequences. See Table 1 for our approach to data analysis in the current study.

**Table 1:** Guiding questions used during the data analysis phase

Category	Guiding questions
Surrogate terms	Which words mean the same thing? Is this word/term referring to trustful relationships between HCPs and children?
Related concepts	Does this word/term hold any relationship with trustful relationships between HCPs and children?
Attributes	What are the defining characteristics associated with trustful relationships between HCPs and children?
Antecedents	What are the situations, events or phenomena preceding trustful relationships between HCPs and children? What happens before the establishment of trustful relationships between HCPs and children?
Consequences	What happens after the establishment of trustful relationships between HCPs and children? What happens as a result of trustful relationships between HCPs and children?

According to Rodgers, researchers must work diligently to identify relevant data during the analysis phase, keeping related guiding questions in mind. To guide the data analysis process, questions were formulated for each category (Table 1). This process was fully completed for all papers before we conducted formal analysis. Delaying the analysis in this way helped avoid premature closure or “jumping to conclusions” [13] and minimized the impact of personal bias. Each category of data was examined separately, and the data was organized and re-organized to identify a consensus in the literature. In this way, we identified predominant themes within the categories. Articles were re-read to seek further clarification when necessary.

In an attempt to ensure objectivity, validity, and reduction of bias, the analysis was conducted by first (RD) and second author (MB). The findings were then discussed with the entire team. All authors brought diverse perspectives to the project, with the team comprising a medical student, a pediatric nurse, and two pediatricians, each contributing their unique expertise to enhance the study’s depth and reliability, thereby bringing a variety of experiences (clinical, educational, and academic).

## Findings

**Fig. 2:**
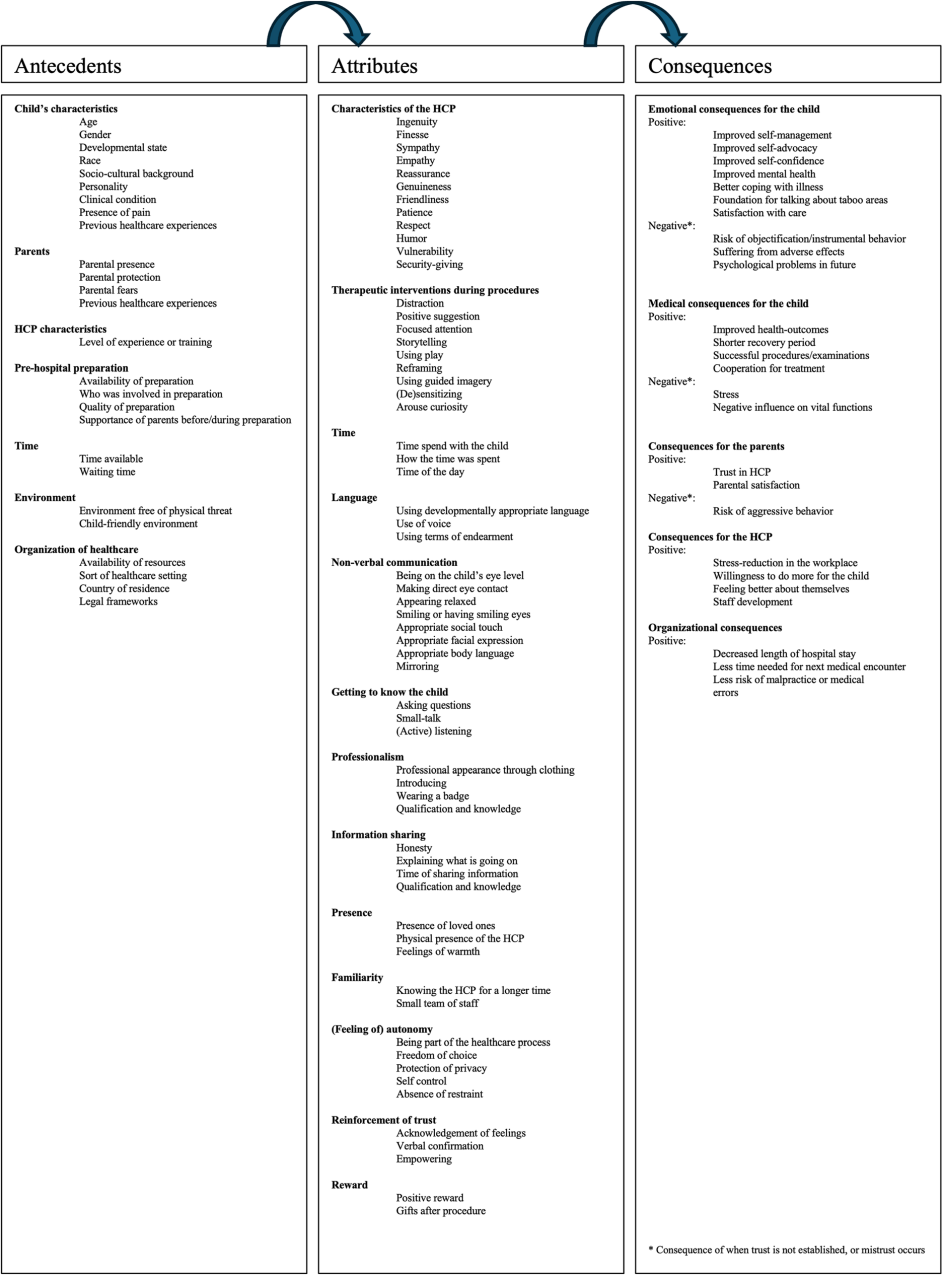
Summary of the results

Rodgers suggests that important concepts are used frequently. Such extensive use can result in the emergence of multiple definitions and interpretations, leading to ambiguity or vagueness over time. Based on our analysis, we identified various ways in which the concept of trust appears in the literature (Fig. 2). What follows is a more detailed discussion of terms relevant to the concept, including (1) surrogate terms, (2) related concepts, (3) attributes, (4) antecedents, and (5) consequences [21].

## Surrogate terms

Surrogate terms are alternative words or phrases used interchangeably to refer to the concept under scrutiny [13]. Of 40 studies, all used the word “trust,” seven articles used the terminology “trusting relationship” [10, 14–19], and one used “trust relationship” [22]. Nine articles referred to using “establishing rapport” or “established rapport” [11, 14, 23–29].

## Related concepts

Related concepts are “concepts that bear some relationship to the concept of interest but do not seem to share the same set of attributes” [13]. Examples of such concepts included “child-centered care” [7, 9, 14, 30], “therapeutic relationship” [31, 32], “patient-oriented care” [33], “connection” [34, 35], “partnership” [25, 36], “doctor-patient relationship” [37–40], “empowering relationship” [10], or “collaborative relationship” [7]. These more broadly used terms appear to imply trust.

## Attributes of a trustful relationships

By closely examining the most common uses of a concept, we can identify its attributes, clarify our understanding of the concept [21], and better recognize instances when the concept of interest is used [13]. Based on our analysis, we identified 13 themes with a total of 61 codes. Although we will discuss the attributes individually, we note that these themes are not mutually exclusive given the overlap in their presentation in the literature. We view them as a “best-fit” in the context of how they emerged overall in the literature reviewed.

### Characteristics of the HCP

We identified numerous characteristics of the HCP as important attributes related to trustful relationships with children during medical encounters. Overall, these characteristics highlighted the importance of HCP genuineness [24], sympathy [14, 15, 31], and friendliness [11, 24, 35, 41] to engage with children in an authentic way. One essential attribute was being respectful [7, 9, 11, 15, 18, 30, 32, 34], for example, Lin presented respect as treating children equal to their parents [32], while Davison added that taking the child seriously indicated respect [9]. Other characteristics included vulnerability [11, 17, 31], security-giving [18, 24, 31, 32, 35, 36, 41–43], and using humor [9, 11, 18, 35, 36, 44–46].

### Therapeutic interventions during procedures

Therapeutic interventions we identified pertaining to trust included attributes such as distraction [11, 17, 28, 29, 31, 36, 41, 47], positive suggestion [11], storytelling [11, 23, 29], and play [7, 11, 17, 18, 22, 26, 27, 33, 35, 36, 39, 41, 44, 46, 48]. The attribute of (de)sensitization was also frequently mentioned [22, 33, 34, 44, 48], involving the gradual introduction of the child to a medical object or HCP in a controlled and positive manner, thereby increasing the child’s comfort and reducing their anxiety.

### Time

Time was a crucial attribute, encompassing both the duration of the physical time that the HCP was willing to make for the child [9, 11, 18, 32, 33, 38, 41, 44], as well as how well this time was spent [49]. Worobey et al. stated that “it is not the time spent with a patient that is seen as important but how much the physician concentrates on the patient during that time” [49 (p293)]. In addition, the literature reviewed referenced the potential impact of the time of day on a child’s behavior and the length of an appointment, which should be adjusted to the child’s age [16].

### Language

Attributes related to language and verbal communication included using developmentally appropriate language [9, 14, 15, 23, 25, 27, 32, 35, 36, 44, 45, 49], modulating voice [11, 15, 17, 28, 34, 36, 39, 40, 45, 48, 49], and using terms of endearment [11, 16, 27, 36, 49, 50]. The use of voice served as an “instrument of calming” to establish trust with a child [48 (p387)], with a soft, kind, but clear tone [17, 34, 36, 40]. Terms of endearment to engender trust were presented in a number of ways in the literature with some suggesting the HCP should learn and use the child’s preferred name or nickname [11, 16, 27, 49, 50], while one article suggested using specific terms such as “sweetie pie” or “darling” [36].

### Non-verbal communication

We identified a number of non-verbal communications with specific importance for HCPs in developing a trusting relationship with a child. These included being on the child’s eye level [11, 14, 17, 23, 36, 39, 44], making direct eye contact [14, 39, 40], although Singh stated that looking directly into the eyes of a child can cause distrust [39]; appearing relaxed [14, 18, 26, 37, 39]; smiling or having smiling eyes [26, 36, 40, 46, 48]; using appropriate social touch [36, 40, 45]; and maintaining appropriate facial expressions [15, 29, 36, 39] and body language [14, 36, 39, 40]. In relation to both facial and body expression, it is noted that it should be calm [39], reassuring [15], and relaxed [14]. It was also found that mirroring the child’s body posture is of great importance in establishing trust, described as “using a similar posture, language, and vocal tone to communicate in a manner congruent with the other person (child)” [11 (p617)].

### Getting to know the child

Another important theme that emerged from the literature is the importance of truly getting to know the child. This involves more than simply collecting medical information; it means showing genuine interest in the child’s personality, preferences, and emotions. Several studies emphasize the value of asking children personal questions to engage them in conversation and build rapport [14, 36, 39]. Others highlight the role of small talk—casual, friendly conversation—as a way to create a comfortable and familiar atmosphere [9, 23, 31, 45]. Active listening also stands out as a key component [7, 9, 11, 14–16, 18, 35, 36, 39, 44].

### Professionalism

Professionalism of HCPs, demonstrated through their dress code and appearance, could be an important factor in influencing trust with children. Some articles indicated a preference for HCPs to wear professional attire [18, 36, 45, 47], which is neat, clean, and hygienic [36]; smartly [47]; and traditional [18, 45], while others found suggested that a child-friendly outfit, like casual clothing [37] or colorful uniforms [35], is preferred. Additionally, professionalism to engender trust was found to include wearing a name badge and introducing oneself [11, 16, 24, 27, 36, 40, 41, 45], as well as through the qualifications and knowledge of the physician [9, 10, 18, 24, 32, 36, 38].

### Information sharing

Information sharing was an important theme in the concept of trust, which includes honesty [7, 9, 11, 16, 18, 32, 35, 36, 38, 44], explaining to the child what is going on [7, 16, 18, 22, 34, 35, 39, 41, 44, 45, 47, 50], as well as the time of sharing information [7]. For example, Coyne et al. detailed the impact of the timing of information on the child’s frame of mind [7].

### Presence

Presence was described as both physical [7, 9, 15, 23–25, 32, 35, 40, 42, 43, 48, 50] and emotional [9, 24, 35, 45, 50]. This was predominantly in relation to the company of loved ones supporting the child to trust in the care delivered, with mothers frequently mentioned specifically as the preferred person to have present. The importance of the physical and emotional presence of the HCP was also valued in terms of their companionship [9, 15, 32, 34, 36, 41], thereby engendering trust.

### Familiarity

The literature highlighted that a relationship between a child and a HCP strengthens over time [18], especially if the child had repeated visits to the same team over a period of time [9, 18, 32, 35, 43, 45]. This would more likely happen in a smaller team: “care should be provided by a small team of professionals so that each child and parent is familiar with those responsible for their care” [35 (p191)].

### (Feeling of) autonomy

Authors ascribed great importance to elements of autonomy. Children should participate in healthcare processes or decision making [7, 9, 10, 14, 18, 24, 32, 35, 40, 41, 43, 44, 47], exercise freedom of choice [17, 32–35, 42], and be ensured that their privacy will be protected [16, 23, 35, 43]. The loss of self-control increases distress and fear, causing distrust [9, 27, 28, 32–34, 42–44, 47, 50]. The impact of loss of self-control on the child’s autonomy featured prominently in the restraint literature [28, 33]. For example, Armfield and Heaton found that restraint is stated to be controversial and inhumane and unacceptable unless “the situation is potentially life-threatening” [28 (p402)].

### Reinforcement of trust

We identified multiple attributes from the literature that highlight the importance of acknowledging [9, 11, 15, 16, 23, 24, 34, 35, 38, 43, 44, 47], confirming [15, 16, 23, 24], and empowering [15, 16, 23, 24, 32, 34–36, 44, 47] the feelings of the child. For example, Boggs and Eyberg wrote in their 1990 book on establishing rapport that acknowledgements are “intended to recognize the child’s efforts, express empathy, or provide feedback to the child that the interviewer (HCP) is listening and understanding” [24 (p87)]. Later research amplified this theme: “feelings do not disappear if ignored” [47 (p31)].

### Reward

Reward implied both verbal positive reward [24] and gifts for the child after the medical visit or procedure [41, 47]. A suitable gift could be a sticker or a toy [47]. Sjöberg et al. stated that gifts could also be given before children experience something unpleasant, as encouragement [41].

## Antecedents of a trustful relationship

Antecedents of a concept refer to the events or conditions that occur before the concept arises or becomes evident. Antecedents may include individual or environmental factors, behaviors, experiences, or circumstances that contribute to the development of the concept [13]. Understanding the antecedents of a concept can help to inform the design of interventions or strategies aimed at preventing or managing the concept, as well as to identify risk factors or potential triggers. We identified seven themes, with a total of 26 codes. Each theme will be presented individually with some overlap in places, similar to the attributes.

### Child’s characteristics

Characteristics of the child, in the context of trust, contribute to how the relationship with a HCP likely develops. Some of these characteristics may not be possible to influence, and therefore, a HCP has to take into account the potential impact of these traits on the child’s ability and willingness to develop a trusting relationship with the HCP. Examples of factors that may all have an impact on the child’s capacity to establish trust included the child’s age [10, 11, 14, 17, 18, 24, 27, 31, 33, 44, 45], developmental stage [7, 10, 11, 17, 24, 31–33, 38, 44, 46, 51], gender [11], race [19], or socio-cultural background [7, 14, 38, 40, 44]. In addition, the child’s clinical condition [7, 18, 32, 44] and the presence of pain [17, 18], as well as personality [7, 10, 14, 23] and prior experiences in healthcare [10, 14, 16, 18, 28, 29, 33–36, 41–43, 45], may all had an impact on the child’s capacity to establish trust.

### Parents

Antecedents related to parents found in the literature included parental presence [7, 9, 15, 23–25, 32, 35, 40, 42, 43, 48, 50], parental protection [9, 32], and parental fears and past medical experiences [11, 14, 16, 28, 29, 31, 34, 36, 37, 44, 50]. Parental experiences referred to the significant impact of the past experiences of parent(s) and caregivers, both positive and negative, on whether the child ultimately builds a trusting relationship with a HCP. This can be impacted by parents’ own fears [11, 16, 37], stress [50], and also from, for example, their own childhood experiences [34].

### HCP characteristics

Compared with numerous characteristics of children, we found little in the literature about HCP characteristics beyond level of experience or training [10, 24, 29, 38, 44, 51]. Boggs and Eyberg stated that the skill of building rapport can only be learned through direct training and experience [24]. However, more recent papers highlight that this remained an issue, with some stating that HCPs continue to receive very little training on how to communicate with children and therefore are less competent in the skill of building relationships [10, 44], whereas others preferred developing this skill over decades, through clinical experience, instead of academic training [29].

### Pre-hospital preparation

An important theme in the antecedents of building trustful relationships was preparation of the child before hospital or procedure. This included discussion in the literature on the availability of preparation [7, 16, 18, 22–24, 31, 33, 41, 44, 47], the people involved in the preparation [16, 33], and the quality of the preparation [7, 33, 41]. For example, Chan, in 1980, stated that the preparation was best performed by all personnel caring for the child, including parents, though it was acknowledged then, and continues to be the case, that parents often needed help in understanding the importance of preparation and the implementation of it [16, 18, 33, 43].

### Time

We identified time as an antecedent in relation to two factors: (a) the often limited time available in the healthcare system to establish trust [18, 32, 33, 38, 41, 44] and (b) the waiting time before consultation or procedure [18, 41, 45]. Sjöberg found that waiting before surgery could result in tension, fears, and anxiety [41], whereas Sheehan and Feely stated that besides procedures, the wait for test results, pain relief, and food were among the worst experiences for hospitalized children [18].

### Environment

The clinical care environment appears to be important in establishing trust. Antecedents included an environment free of physical threat [18, 23, 39, 45, 50], such as visible medical instruments [23, 45, 50], unpleasant sounds [39, 45], and a child-friendly environment [11, 39, 41, 45, 47], for example, with color [45, 47], toys to play with [41, 45], and books [45].

### Organization of healthcare

One of the antecedents of this theme was availability of resources to care for the child [10, 38, 44]. Examples included a well-resourced clinical area that had the timely capacity to offer skilled care, sufficient clinical staff, and the availability of hospital budget to pay for certain services [10, 44], but also lack of supervisory feedback on residents’ performance [38]. The ability of a child to develop a trustful relationship was also found to be influenced by the specific area of healthcare in which the child was cared for. O’Neill stated that there is often too little time to develop a trustful relationship in critical care settings, whereas there may be more opportunity in primary care, where the HCP could develop a bond over time [14]. Another antecedent in this theme was country of residence. Lin et al. stated “Participants (hospitalized children), particularly those from China and Taiwan, felt clinicians were ‘colluding’ with parents to hide their diagnosis, which may relate to cultural values pertaining to child autonomy and parental protection” [32]. Another aspect of healthcare is the legal framework in which HCPs must act [10, 44, 51] and the attention given to children’s rights in healthcare. For example, articles referred to the United Nations Convention on the Rights of the Child (2) and the Child and Adolescent Health Strategy for Europe [44].

## Consequences of a trustful relationship

Identifying the consequences of a concept further enhances understanding of the topic of interest [14]. From the literature reviewed, five themes emerged, with a total of 26 codes.

Similar to the section on attributes and antecedents above, these themes are not mutually exclusive; rather, they are a “best-fit” in the context in which we identified them in the literature reviewed.

### Emotional consequences for the child

The impact of sickness, hospitalization, and procedures on the emotional and mental wellbeing of the child is well-known. Almost all articles suggested that the presence of trust positively influences the emotional state of the child. Children experienced improved self-management [43], self-advocacy [9], self-confidence [41], and improved mental health [7], when a trusting rapport is established. Pérez-Duarte Mendiola stated that it can help children to accept and cope with their illness [44], while Damm et al. suggested that the trustful relationship can be the foundation for talking about difficult or taboo areas [10]. All of this has the potential to increase the satisfaction of the child with their experience of care [7, 9, 14, 18, 22, 24, 30, 34, 39]. When trust was not established, there is a risk of objectification or instrumental behavior [9, 10, 40, 50]. By this, we mean that the child is referred to as an object, rather than an autonomous individual with feelings and perspectives. Damm et al. outlined in their article that “if the pediatric team has not learned how to handle difficult situations and to build relationships of trust and empowerment … they will talk about difficult children as if they were objects instead of with them as members of a team” [10 (p1328)]. Furthermore, Bari et al. stated that when trust was not established, the majority of HCPs in their study had “…treated the patient as a medical object and ignored them as a human being. The main aim of their communication was to reach a right diagnosis” [40 (p1315)]. When distrust occurred, children can later suffer from adverse effects, including fear, sadness, anxiety, loneliness, and feelings of being rejected [9, 29, 41, 44]. Skipper and Leonard stated in their article that even psychological problems in later life can occur [50].

### Medical consequences for the child

Medical consequences of trust referred to improved health outcomes [7, 18, 26, 29, 35, 39, 44], shorter recovery period [49, 50], successful procedures or examinations [29, 31, 39], and cooperation for treatment [7, 9–11, 14, 15, 18, 32, 35, 39, 44]. When trust was not established between the HCP and the child, stress could occur, which can result in negative vital functions, like elevated temperature, pulse rate, and blood pressure [50].

### Consequences for the parents

Trustful relationships between children and HCPs could have positive influences on parent(s) or caregiver(s). When children show trust in their HCPs, parental satisfaction improved [14, 30, 33, 39] and parents were often more likely to trust their child’s HCP themselves [39]. Without trust, there may be a greater risk of aggressive behavior of the parent towards the HCP [18, 39].

### Consequences for the HCP

HCPs also experienced positive consequences of the trustful bond between himself and the child. For example, Boggs and Eyberg state that when trust is formed, the HCP is willing to do more for a child [24]. It could also make the HCP feel better about themselves and overall could have a positive impact on stress levels and staff development [34].

### Organizational consequences

Organizational consequences refer to the impact that a trustful relationship between the child and HCP may have on the healthcare organization. Time seems to play a part in this, as Singh stated that the presence of trust could decrease the length of a child’s hospital stay [39] and Damm et al. found out that when a trusting bond between the child and the HCP is established, there is less time needed for the next medical encounter between this HCP and child [10]. It was also found that the risk of malpractice or medical errors decreases when there is a relationship of trust [14, 39, 40].

## An exemplar of the concept of a trustful relationship

An example of the concept of a trustful relationship, both from literature and practice, can give us more clarity about how a concept appears in everyday life. It is important to be aware of the risks of bias when constructing. For this reason, Rodgers recommends using an example from literature. Although Rodgers suggests the inclusion of an exemplar of the concept, no clear exemplar emerged from the literature which reflects all the key characteristics of the overall concept of a trustful relationship.

## Discussion

This study explored the concept of trustful relationships in pediatric healthcare. Our findings illustrate that trust is not a static or singular construct, but an evolving and multifaceted phenomenon. It is shaped by HCPs’ characteristics and behaviors, such as empathy, genuineness, and respect, alongside concrete practices like therapeutic play, age-appropriate language, and attentive non-verbal communication. Trust is further influenced by children’s prior healthcare experiences, their emotional state, and the healthcare environment itself. Pediatric trust-building differs substantially from adult healthcare relationships. Children’s heightened sensitivity to relational and environmental cues, such as playfulness, tone of voice, and body language, necessitates HCPs to engage in developmentally attuned and emotionally present care [52–54]. When trust is successfully established, outcomes include improved emotional well-being, greater cooperation during encounters, enhanced medical results, and increased satisfaction among children and parents.

Based on our systematic literature review and analysis, we propose defining trustful relationships between children and HCPs as “dynamic, reciprocal connections characterized by mutual curiosity, respect, and confidence. These relationships form the foundation of effective clinical care and develop through the interplay of contextual factors and professional behaviors intended to establish trust.” We further posit that these relationships require specific competencies in age-appropriate communication and interpersonal skills that meaningfully engage and empower children in their healthcare experience [55, 56].

These findings align with key theoretical frameworks that have been related to trustful pediatric care: *Erikson’s developmental theory* positions trust as the foundation of healthy psychological development [57], while *social cognitive theory* explains how children develop trust through observation and modelling. *Self-determination theory* highlights the role of autonomy, relatedness, and competence in fostering intrinsic motivation, and *humanized care theory* emphasizes dignity and personhood in healthcare encounters [8, 58].

We acknowledge that this definition should be considered preliminary, as our study represents an initial attempt to describe trustful relationships as a distinct concept within pediatric healthcare. Nevertheless, having an empirically grounded definition provides a valuable framework for future scientific exploration and further theoretical development [13]. Moreover, it offers a foundation for developing quality outcome measures in pediatric care [59] and designing evidence-based communication training programs for healthcare professionals working with children [6, 60, 61].

Despite its significance, trust remains underrepresented in pediatric healthcare education and policy making. The American Academy of Pediatrics’ policy statement on Family-Centered Care mentions trust only once [62], and the Dutch pediatric training framework does so twice, without elaboration [63]. This limited attention in formal policy documents highlights a critical misalignment between clinical priorities and educational standards.

Trust is often assumed to arise naturally, yet our findings show that specific, teachable competencies are required. Trust-building must be recognized as a core clinical competency, not left to intuition or experience alone. Educational programs should therefore integrate structured, developmentally attuned communication and relationship-building training that reflect the emotional, cognitive, and social capacities of children. This includes investment in realistic pediatric simulations and continuous professional development across developmental stages. Ideally, trust-building strategies should be integrated early and also in conjunction with the teaching of potentially distressing physical examination skills (e.g., ear and throat exam) as well as procedural skills (e.g., venipuncture, urinary catheterization). Innovative approaches have integrated interactions with simulated parents on the one hand and authentic interactions with children into such curricula [64]. In addition to educational efforts, clinical environments should be intentionally designed to foster trust, through child-friendly spaces, privacy considerations, and emotionally safe atmospheres. Finally, the development of assessment tools to evaluate the quality of trustful relationships between children and healthcare professionals could provide valuable metrics for care quality and support evidence-based improvement.

The integration of trust-building competencies into pediatric healthcare represents not merely an educational and architectural enhancement, but a fundamental shift toward recognizing children as active participants in their healthcare experience. This transformation requires coordinated efforts across educational institutions, healthcare organizations, and policy-making bodies to ensure that the next generation of healthcare professionals is equipped with the skills necessary to establish meaningful, trustful relationships with their youngest patients.

## Strengths

By addressing the conceptual gap in the literature on trustful relationships in pediatric healthcare, this study contributes to the theoretical development of child-centered care and lays important groundwork for future research and practical applications. Another key strength of this study is the multidisciplinary composition of our author team, each bringing unique perspectives to the concept of trustful relationships. This diversity of expertise has enriched the analysis and interpretation of findings, contributing to a more nuanced understanding of trust in pediatric healthcare.

## Limitations

Despite our comprehensive search strategy, we may have missed relevant studies on trust in pediatric healthcare, particularly those employing different terminology or emphasizing related but distinct aspects of trust. In addition, utilizing broader search parameters, including studies focused on older children, children with intellectual disabilities, and those with mental health disorders, might uncover additional insights not captured in our review. Second, our study maintained an exclusive focus on the HCP relationship without adequately considering the vital triadic dynamic that includes parents. Since parental trust and emotions significantly influence children’s healthcare experiences, understanding the complex interplay of trust between parents and HCPs represents an area requiring further exploration. Finally, most cited researchers, as well as our author team, have predominantly Western backgrounds. Given that our findings indicate trustful relationships are deeply shaped by cultural contexts, including communication patterns, power dynamics, family involvement, perceptions of childhood, and language barriers, this represents a significant limitation in the generalizability of our findings.

## Future research directions

Given the emergent nature of trustful relationships as a distinct concept in pediatric healthcare, we recommend scholars and practitioners to start a dialogue to define and refine the concept of “trustful relationships."Several avenues for future investigation should be considered. Further studies are needed to systematically explore how trustful relationships vary across different developmental stages (from infancy through adolescence) and healthcare settings (acute versus chronic care, inpatient versus outpatient environments). Mixed-methods approaches combining observational studies with child-centered interview techniques would be particularly valuable for capturing the child’s perspective on trust formation processes. Longitudinal studies examining how trust evolves over time and across multiple healthcare encounters would significantly enhance our understanding of this dynamic relationship. Intervention studies testing specific trust-building or trust-repairing approaches are essential for translating theoretical understanding into evidence-based practice. Finally, future research must prioritize cross-cultural investigations to understand how diverse cultural contexts shape trust in pediatric healthcare. This includes examining non-Western healthcare systems, minority populations within Western contexts, and indigenous healthcare practices to develop a more comprehensive and globally applicable understanding of pediatric trust relationships.

## Conclusion

This concept analysis unveils crucial insights into fostering “trustful relationships between healthcare professionals and children” in clinical settings. Through Rodgers’ evolutionary approach, we identified key attributes, antecedents, and consequences of trust-based interactions. The findings illuminate the relevance for specific, teachable competencies. Incorporating these insights into clinical education promises to transform patient-provider dynamics, ultimately enhancing treatment efficacy and compassionate care delivery. Further research should expand upon these dimensions to deepen understanding and application across diverse healthcare settings.

## Supplementary Information

Below is the link to the electronic supplementary material.

Supplementary file1 (PDF 222 KB)

## Data Availability

No datasets were generated or analysed during the current study.

## Abbreviations

CA: Concept analysis
ECA: Evolutionary concept analysis
HCP: Healthcare professionals
