# Supplementary material for: Trustful relationships between healthcare professionals and children: a concept analysis using Rodgers’ evolutionary approach
Source: Eur J Pediatr. 2025 Jul 3;184(7):464. doi: 10.1007/s00431-025-06297-0 (PMC12222351; doi:10.1007/s00431-025-06297-0)
Supplement: Supplementary file 1 — Supplementary file1 (PDF 222 KB) [file 431_2025_6297_MOESM1_ESM.pdf]

Supplementary materials:

Characteristics of articles included in the concept analysis

Trustful relationships between healthcare professionals and children: A Concept Analysis using Rodgers' Evolutionary Approach

Rhymme Dickens[1] • Piet Leroy[1] • Walter Eppich[2] • Maria Brenner[3]

Rhymme Dickens
rhymme.dickens@maastrichtuniversity.nl

Piet Leroy
p.leroy@mumc.nl

Walter Eppich
w.eppich@unimelb.edu.au

Maria Brenner
maria.brenner1@ucd.ie

[1] School of Health Professions Education, Faculty of Health, Medicine and Life Sciences, Maastricht University, Maastricht, the Netherlands

[2] Faculty of Medicine, Dentistry and Health Sciences, University of Melbourne, Victoria 3010, Australia

[3] School of Nursing, Midwifery and Health Systems, University College Dublin, Health Sciences Building, Belfeld Dublin 4, Ireland

Authors	Year	Country	Study design	Aim of paper	Key conclusions
**1.** Appel & Strecker	1936	USA	Chapter, book	To inform about developing rapport in child psychology to obtain crucial information about a child's personality and underlying issues during medical examinations, especially when the child is apprehensive or unaware of the purpose of the visit.	Establishing a secure, trusting relationship and carefully observing the child's behavior and responses are crucial for effectively understanding and addressing their underlying needs in a clinical setting.
**2.** Skipper & Leonard	1968	USA	Randomized experimental study	To investigate whether reducing maternal stress through enhanced social interaction with HCPs can indirectly decrease stress in young children undergoing tonsillectomy.	Reducing maternal stress levels can significantly reduce stress in young children undergoing tonsillectomy, leading to better physiological, psychological, and social outcomes.
**3.** Chan, J. M.	1980	USA	Chapter, book	To explain how play techniques used by child life therapists can help hospitalized children prepare for and cope with medical procedures and surgery, and enhance their understanding and trust in HCPs.	Psychological preparation through play significantly helps hospitalized children cope better with medical procedures, fosters their cooperation, and increases their trust in HCPs.
**4.** Fosson & deQuan	1984	USA	Descriptive approach	To provide a structured approach for HCPs to effectively communicate with seriously ill or hospitalized children, aiming to alleviate their anxiety, validate their feelings, and offer reassurance through various communication strategies.	Using a systematic approach to communication, including labelling, idioms, storytelling, and reframing, can significantly enhance the ability of HCPs to comfort and support hospitalized children and their families.
**5.** Worobey et al.	1984	USA		To analyze and describe the communication interactions between pediatricians, patients, and parents, specifically examining how pediatricians adjust their speech patterns to fulfil different roles in the pediatric triadic (child-parent-HCP) relationship.	Pediatricians adjust their communication style, using friendly, gentle authority, and consultative roles to effectively address the differing needs of children and parents, thereby enhancing both emotional comfort and perceived competence during medical interactions.
**6.** Boggs & Eyberg	1990	USA	Chapter, book	To provide comprehensive guidance on effective communication skills and strategies for establishing rapport with children and adolescents.	To successfully engage with a child, a HCP needs communication skills and interviewing strategies. These skills include acknowledgement, reflecting, praising, questioning and summarizing

**7.**	Blazys, D.	1999	USA	Opinion paper	Opinion piece on providing practical strategies for gaining the trust of children in the emergency department.	Key factors include using playful interactions, involving parents in the care process, and employing calming techniques such as soothing voices and gradual approaches to build trust and reduce anxiety in children in the emergency department.
**8.**	Tabakman, L.	1999	Canada	Case study	To demonstrate how establishing trust and incorporating compassionate, playful, and patient-centered techniques in dental practice can improve children's experiences in the dental office.	Implementing trust-building techniques, respect, and compassionate care in dental practice leads to improved patient experiences and outcomes, fostering positive relationships and reducing anxiety for both children and parents.
**9.**	O'Neill, K. A.	2002	USA	Practice guideline	To provide HCPs with strategies for effective communication with school-aged and adolescent patients and their families in acute care settings.	Effective communication in acute care settings, is characterized by active listening, understanding, and involving both the child and the parent in decision-making. This improves patient satisfaction, care quality, and can help reduce malpractice risks.
**10.**	Nash, D. A.	2006	USA	Review	To review and highlight the importance of communication skills essential for pediatric dentists to establish effective and empathic relationships with child patients.	reflective listening, self-disclosing assertiveness, and descriptive praise are critical communication skills that pediatric dentists should use to foster a positive, empathetic, and cooperative relationship.
**11.**	Pelander et al.	2007	Finland	Cross-sectional survey	To evaluate the quality of pediatric nursing care from the perspective of Finnish children aged 7 to 11 by assessing their expectations and experiences of hospital admission.	While Finnish children generally rated the quality of pediatric nursing care positively, there were notable areas for improvement, particularly in supporting children's autonomy, providing engaging activities, and ensuring clear communication and information tailored to their developmental needs.
**12.**	Brady, M.	2009	United Kingdom	Qualitative research	To explore and identify the characteristics of a good nurse from the perspective of hospitalized children between 7 and 12, from Southeast England, addressing a gap in the	Hospitalized children value nurses who are competent, understanding, trustworthy, truthful, and attentive. Who demonstrates effective communication and where fun.

				literature and informing children's nursing practice.	
**13.** Plumridge et al.	2009	New Zealand	Conversation analysis research	To examine the elements of partnership and communication between nurses and parents during events of immunization.	The use of small talk and 'baby-talk' by nurses during immunizations plays a crucial role in managing the child's distress and maintaining effective communication with child and parents.
**14.** Singh, M	2009	India	Opinion paper	To emphasize the importance of a holistic and comprehensive approach to pediatric care to enhance the overall well-being and development of children.	Pediatric care requires a holistic approach, focusing on the health, nutrition, education, and emotional support of children and their families.
**15.** Golden, S.	2010	USA	Chapter, book	General guideline for HCPs for how to talk with children during the medical interview.	Children can be reliable reporters of their experiences when interviewed by a skilled interviewer who understands their developmental strengths and limitations, as long as the interviewer remains attentive to the child's unique communication style, uses simple language, and avoids assumptions about the child's knowledge or perspective.
**16.** Nilsson et al.	2011	Sweden	Qualitative content analysis	A report of the experiences of children (5–10 years) of procedural pain when they underwent a trauma wound care session.	Children need to experience clinical competence, distraction, security and participation, in order to be able to trust the HCP to carry out the wound care.
**17.** Salmela et al.	2011	Finland	Semi-structured interview	To explore and describe the subjective experiences of hospital-related fears in 4- to 6-year-old children in Finland.	Hospital-related fears in pre-school-aged children can lead to significant emotional and developmental impacts, including insecurity, decreased self-esteem, and mistrust of healthcare professionals. These fears can be alleviated through proper preparation, parental support, and positive experiences in the hospital setting.
**18.** Hofmann et al.	2012	Austria	Semi-structured questionnaire	To determine whether different clothing styles of pediatricians influence the opinions of children and parents regarding the doctor, and to identify which outfit is preferred by both groups.	Both parents and school-aged children prefer pediatricians in casual outfits, which do not diminish trust, while clothing style has no significant impact on the behavior of children under six years old.

**19.**	Armfield & Heaton	2013	Australia	Review	To review and provide practical advice on non-pharmacological techniques for managing dental anxiety in patients, including children.	Managing dental anxiety in children is achievable through a tailored, non-pharmacological approach that includes providing information, reinforcement and distraction.
**20.**	Lowe, O.	2013	USA	Review	To emphasize the importance of effective communication between dentists, parents, and children in ensuring successful pediatric dental care, focusing on building rapport, managing expectations, and fostering positive dental experiences	Effective communication between the dentist, parents, and child, including building rapport, managing expectations, and involving parents in the process, is crucial for successful pediatric dental care and achieving positive treatment outcomes.
**21.**	Mastro et al.	2014	USA	Integrative literature review	To summaries findings from an integrative literature review on patient- and family-centered care (PFCC) in children's hospitals, providing evidence-based recommendations for nurse executives to effectively implement PFCC in clinical practice.	While evidence supporting patient- and family-centered care (PFCC) is still emerging, it suggests benefits in patient and staff satisfaction, and emphasizes the need for nurse executives to support and implement PFCC principles by developing essential competencies and evaluating their impact on outcomes.
**22.**	Damm et al.	2015	Austria	Opinion paper	To explore how pediatric consultations often marginalize children by focusing communication primarily on parents and doctors, and to advocate for better integration of children's perspectives in medical discussions to enhance their empowerment and participation in their own healthcare.	It is crucial for pediatricians to receive specialized training in child-friendly communication to better involve and empower children in their own healthcare.
**23.**	Damm et al.	2015	Austria	Opinion paper	To highlight the need for better communication between pediatricians and children, advocating for improved training and integration of child-friendly practices in healthcare.	Effective communication between pediatricians and children is crucial for enhancing the child's competence and autonomy, necessitating targeted training for healthcare providers to improve child-centered care and respect for children's rights.
**24.**	Jullien et al.	2015	France	Practical intervention study	To evaluate the effectiveness of a preoperative learning-through-play protocol using Playmobil® kits and an equipped doll to reduce anxiety in children undergoing cardiac surgery.	Using toys for preoperative education significantly reduces anxiety in children undergoing cardiac surgery and improves overall satisfaction among families and healthcare providers.

**25.** Moore, A.	2015	Ireland	Practical guide	To provide a comprehensive, step-by-step approach for managing anxiety in children during dental visits by employing strategies to build trust, comfort, and cooperation.	Recognizing and addressing a child's anxiety through communication, supportive techniques, and a reassuring environment can significantly improve their comfort and cooperation during dental visits.
**26.** Sjöberg et al.	2015	Sweden	Narrative interviews and Qualitative content analysis	To describe the experiences of participation in perioperative care of 8- to 11-year-old children in Sweden	Improving children's participation in perioperative care involves providing more preparatory information, adapting the care environment to their needs, reducing waiting times, and enhancing communication and decision-making involvement to reduce anxiety and improve their overall experience.
**27.** Alofisan et al.	2016	Saudi Arabia	Cross-sectional research	To assess pediatric residents' attitudes towards communication skills, their perception of the importance of these skills, and their confidence in utilizing them during their medical training in Saudi Arabia.	while pediatric residents in Saudi Arabia recognize the importance of communication skills they lack confidence in handling more complex issues like delivering bad news and discussing end-of-life care, highlighting the need for communication training in residency programs.
**28.** Coyne et al.	2016	Ireland	Descriptive qualitative research	To explore the views and experiences of children, parents, and healthcare professionals regarding children's participation in information-sharing interactions in a children's cancer unit.	Flexible, sensitive information-sharing is essential in triadic encounters, balancing openness with parents' and children's diverse needs.
**29.** Singh, M.	2016	India	Narrative review	To emphasize the importance of effective doctor-parent communication in building trust, improving patient care, and addressing the declining image of the medical profession.	Improving doctor-parent communication and restoring a compassionate, respectful approach in medical practice are essential for rebuilding trust.
**30.** Bari et al.	2017	Pakistan	Qualitative exploratory research	To analyze communication skills of pediatric postgraduate residents from Pakistan in clinical encounter by using video recordings.	While pediatric residents are polite in their communication, they exhibit poor nonverbal skills, treat patients mechanically as medical objects, and largely exclude children from active participation during clinical encounters.
**31.** Holmstrom et al.	2019	Sweden	Qualitative content analysis	To describe nurses' experiences in prehospital care encounters with	Nurses face significant challenges in providing pain relief to children in

				children in pain and the specific related challenges.	prehospital care, requiring them to be mentally, practically, and theoretically prepared, and emphasizing the need for better training, support, and research to ensure effective pediatric pain management.
**32.** Crowe, A.	2020	Ireland	qualitative, exploratory research	To explore the importance of developing effective communication skills in anesthesiology trainees working with children, particularly during anesthesia induction and in the context of challenges posed by face mask wearing during the COVID-19 pandemic.	Effective communication skills in anesthesia are essential for reducing distress in children, and specific training is needed to help trainees develop these skills, especially during mask-wearing.
**33.** Lin et al.	2020	Australia	Systematic review	To describe the experiences of children and adolescents communicating with clinicians during childhood cancer treatment to inform strategies for improving patient-centered communication and decision-making.	Children and adolescents with cancer benefit from respectful, empathetic, and developmentally tailored communication, which enhances their sense of empowerment and control, and improves their overall care and outcomes.
**34.** Mann & Kennedy	2020	Australia	Qualitative research, using semi-structured focus groups and in-depth interviews	To identify which communication techniques used locally by pediatric anesthetic specialists, trainees, and nurses are viewed as the most effective and valuable to teach trainees.	Core communication techniques and principles, such as storytelling and guided imagery, are effective in pediatric anesthesia and recommended incorporating these into trainee education through interactive and experiential learning.
**35.** Sheehan & Fealy	2020	Ireland	Cross-sectional descriptive research	To measure hospitalized children's trust in nurses and analyze the impact of various factors on trust in the nurse.	Children's trust in nurses is positively correlated with adherence to care and less fear, and is influenced by age, prior hospital exposure, and positive nurse interactions, highlighting the importance of fostering trust to improve patient outcomes.
**36.** Davison et al.	2021	United Kingdom	Scoping review with interpretive phenomenology approach	To explore and synthesize children's and adolescents' lived experiences of healthcare professionals by analyzing qualitative accounts to better understand their interactions and perceptions.	children and adolescents value healthcare professionals who build trusting relationships, involve them in decision-making, and communicate effectively, while poor communication and lack of

					involvement can lead to fear and disengagement.
**37.** Perez-Duarte Mendiola, P.	2022	United Kingdom	Qualitative research	To identify strategies and methods used by Health Play Specialists to effectively communicate with hospitalized children and to advocate for the integration of 'play' in pediatric healthcare settings.	Health Play Specialists (HPS) use play-based methods and material resources to enhance communication with hospitalized children and help them understand their illness experiences, emphasizing the need for a 'pro-play' approach and greater recognition of HPS in pediatric care.
**38.** Sarkar et al.	2022	India	Qualitative research	To examine how various communication strategies, including both verbal and nonverbal methods, can be used to improve the dental care experience for children, the elderly, and middle-aged patients, and to identify ways to address their specific needs and concerns effectively.	Using clear, simple explanations, creating a child-friendly environment, and employing reassuring and empathetic communication techniques are essential for reducing anxiety and improving their dental care experience for children.
**39.** Garcia et al.	2023	USA	Scoping review	To identify and map evidence-based communication practices that improve care for Black pediatric patients and their caregivers to promote health equity and counter racism.	Implementing practices in curriculum would ensure consistent application of effective communication strategies to address racism and enhance care for Black pediatric patients.
**40.** Thomson, L.	2023	USA	Theoretical review	To explore how incorporating humor into hypnotherapy with children and adolescents can enhance therapeutic effectiveness.	Integrating humor into hypnotherapy can enhance therapeutic outcomes by strengthening the clinician-child rapport and leveraging laughter's emotional and physiological benefits.
